# Congenital Dislocation of the Hip

**Published:** 1910-12

**Authors:** 


### Reviews of Books.

**Congenital Dislocation of the Hip.** By J. Jackson Clarke, M.B., F.R.C.S. Pp. vi, 92. London : Baillière, Tindall and Cox. 1910.

Books nowadays often contain descriptions of so many different surgical methods that a reader more or less unacquainted with the subject finds it quite impossible to determine which is the best plan to adopt in the particular case under observation.

In this book the case is not so ; the lines laid down by Lorenz are acknowledged and strictly followed. Added to this we have the author's own experience in about forty cases, extending over a period of more than five years. No wonder, then, that the book is full of practical details, and though small gives a fairly complete review of the whole subject.

Too little space has been allotted to diagnosis, considering that the disease is so uncommon except in the clinics of surgical practice at a hospital. Many cases are quite obvious, but others, equally important, often present difficulties, especially when a good X-ray plate cannot be procured.

The author has devised a more simple operation than the open one practised by Lorenz. It has been too recently introduced for a reliable opinion to be formed upon its merits, but it is rational, and bids fair to give good results. It is only adopted in relapsed cases, or in those which come late under observation. The book should prove a very useful monograph.

